# Microglial ramification and redistribution concomitant with the attenuation of choroidal neovascularization by neuroprotectin D1

**Published:** 2013-08-04

**Authors:** Kristopher G. Sheets, Bokkyoo Jun, Yongdong Zhou, Min Zhu, Nicos A. Petasis, William C. Gordon, Nicolas G. Bazan

**Affiliations:** 1 Neuroscience Center of Excellence, Louisiana State University Health Sciences Center, School of Medicine, New Orleans, LA; 2 Department of Chemistry and Loker Hydrocarbon Research Institute, University of Southern California, Los Angeles, CA

## Abstract

**Purpose:**

Neuroprotectin D1 (NPD1) attenuates laser-induced choroidal neovascularization (CNV) when administered intraperitoneally. Due to its lipophilicity and low molecular weight, NPD1 is well suited for topical delivery; thus, we investigated the efficacy of topically applied NPD1 in attenuating CNV. We also examined the effect of NPD1 on the recruitment and activation of microglia surrounding CNV lesions.

**Methods:**

Mice were given laser-induced CNV and treated with NPD1 eye drops. CNV was evaluated by fluorescein leakage using a novel image analysis method and by isolectin B4 immunofluorescence of neovasculature. Microglia; recruitment was assessed by quantification. Using form factor, solidity, convexity, and fractal dimension, microglial activation was quantitatively assessed by two-dimensional, and for the first time, three-dimensional morphology. An ImageJ plugin, 3D Shape, was developed to enable this analysis.

**Results:**

NPD1 attenuated leakage and neovascularization. The proximity of microglia to CNV lesions was significantly closer with NPD1. Consistent with the cellular ramification, microglia in NPD1-treated eyes were larger and exhibited a lower form factor and higher fractal dimension.

**Conclusions:**

Our data show that NPD1 signaling induces a ramified, non-injury-inducing microglial phenotype coincident with attenuation of CNV. Since microglia are crucial participants in neurodegenerative diseases, the discovery that microglia are potential targets of NPD1 signaling warrants further investigation.

## Introduction

Choroidal neovascularization (CNV) rapidly deteriorates visual acuity and is responsible for most vision loss attributed to age-related macular degeneration. The current treatment options for CNV are limited to invasive laser photocoagulation, photodynamic therapy, and intravitreal injections of vascular endothelial growth factor (VEGF) scavengers [1]. Photoreceptor degeneration and CNV have been linked with the subretinal accumulation of microglia [2], but the role of microglia in these pathogenic events is not yet completely understood. Under physiological conditions, microglia are beneficial, releasing neuroprotective and anti-inflammatory factors [3]. In the diseased state, however, microglia are highly activated and appear to play a pathological role [4,5].

Recently, we demonstrated that a novel docosanoid, neuroprotectin D1 (NPD1), attenuates neovascularization and vascular leakage in a laser-induced model of CNV when injected intraperitoneally [6]. We also showed that NPD1 is a potent mediator of neuroprotection and inflammatory resolution [7-9]. Since microglia have the potential to resolve or exacerbate neuroinflammation, in this study, we examined the effect of NDP1 signaling on microglial cells in laser-induced CNV.

## Methods

### Mice

All animal experiments conformed to the Association for Research in Vision and Ophthalmology statement for the use of animals in ophthalmic and vision research and were approved by the Louisiana State University Institutional Animal Care and Use Committee. Male C57Bl/6 mice (8–12 weeks; 25–30 g) were obtained from Charles River Laboratories (Wilmington, MA) and maintained in the LSUHSC animal colony on a 12h:12h light-dark cycle (0600 h on; 1800 h off) with an average in-cage luminance of 20 lux at bedding level. Animals were fed normal mouse chow and supplied with water ad libitum. Twenty-four mice were used to assess retinal bioavailability and 20 mice were used for laser-induced CNV. Mice were anesthetized with ketamine (200 mg/kg) and xylazine (10 mg/kg) before deuterated NPD1 application, laser treatment, and fundus angiography. All mice were sacrificed by cervical dislocation.

### Treatments

Based on our previous results using intraperitoneal (ip) injection [6], NPD1 dosage was targeted at 1 mg per kg eye wet-weight (16 mg average wet-weight) and 16 ng NPD1 was delivered to the eye surface as a 1.6 μl drop (10 ng/μl NPD1 and 9.75% ethanol in saline) via micropipette. Ipsilateral eyes received NPD1, while contralateral eyes served as within-animal controls and received 1.6 μl of vehicle (9.75% ethanol in saline. The treatment solution was prepared and stored as 25 µl single-use aliquots at −20 °C. Treatments were administered rapidly, between 0900 and 1100, without anesthesia, using only gentle restraint. The treatment schedule was 1 h before laser treatment and once daily through 7 days post laser treatment. All information about each animal throughout the experimental procedures was hidden from the investigator performing the laser and NPD1 treatments.

### Retinal bioavailability

The bioavailability of NPD1 in retinal tissue was determined using NPD1 labeled with two deuterium atoms [10]. Mice were anesthetized as above, and one eye per mouse received topical application of 1.5 μl of NPD1-d2 (100 ng/μl in 97.5% ethanol). This application represents a 10-fold higher concentration to ensure adequate detectability via mass spectrometry. To maintain this concentration of NPD1, the ethanol concentration was also 10-fold higher than the treatment levels. Contralateral eyes served as negative controls and received 2.5% methylcellulose to prevent corneal desiccation. Manual blinking of ipsilateral eyes was performed until mice were awake and moving normally. Mice were sacrificed, as above, at 0, 0.25, 0.5, 1, 2, and 4 h post-NPD1-d2 application. Immediately after this, eye surfaces were thoroughly washed and retinal tissue collected. Retinal tissue was extracted using a gentle pulling force applied with curved forceps behind the eye while the corneal surface was incised with a scalpel. NPD1-d2 was quantified in extracted retinal tissue by triple quadrupole tandem mass spectrometry. Selected parent and daughter ion pairs of NPD1-d2 were 361 and 208 m/z, respectively. Raw concentrations are reported as pictogram (pg) NPD1-d2 detected per mg of retinal protein analyzed. Variations in NPD1-d2 mass applied were corrected and normalized to the average retinal protein to determine the percent of applied NPD1-d2 present in each retina.

### Laser choroidal neovascularization

**Figure 1:**
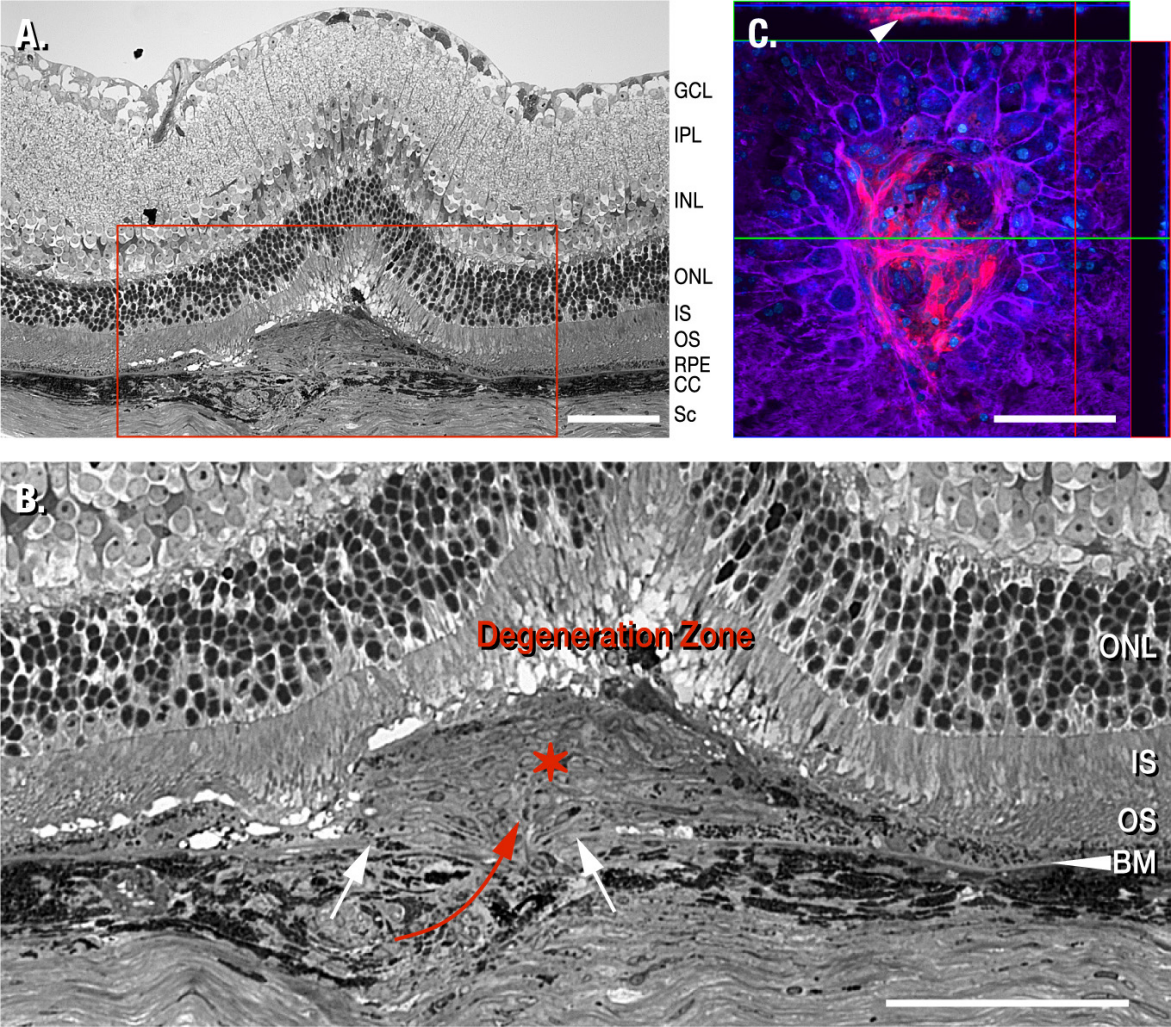
Structure of a laser lesion at 14 days post treatment in a mouse model of laser-induced choroidal neovascularization. **A**: A 1 µm thick plastic section through the center of a lesion was contrasted with toluidine blue. Following initial penetration through the retinal pigment epithelium (RPE) and Bruch’s membrane (BM), inflammatory signaling occurs and vascular endothelial growth factor (VEGF) is upregulated to initialize neovascularization. Vessels grow through the break in BM (denoted by the two white arrows) and ramify between the RPE and the photoreceptor outer segments (red asterisk). **B**: A close-up of the neovascularized region outlined (red box) in A illustrates that BM (white arrowhead) is compromised (space between the white arrows). Newly formed vessels have entered the retina through this gap (red arrow) to spread laterally under the photoreceptors. This mass (red asterisk) displaces the photoreceptors and triggers degeneration (Degeneration Zone). Vessels within this mass originate from within the choriocapillaris (CC), and not from vasculature within the retina. Note that the layers of photoreceptor outer segments (OS), inner segments (IS), and outer nuclear layer (ONL) are continuous. **C**: A confocal image stack of a laser lesion is shown 14 days post treatment. Following removal of the retina, immunohistochemistry was performed on this flat mount eyecup preparation to highlight endothelial cells (isolectin B_4_, red), RPE cells (f-actin, purple), and nuclei (blue). The square box shows a top view of the lesion focused at the surface of the tissue. The horizontal rectangle at the top shows a side view through the image stack at the position of the green line; the vertical rectangle at the right shows a side view through the image stack at the position of the red line. The blue line in both the top and side rectangles shows that the image in the large square is from the top of the image stack. Notice the RPE cells outlined (purple) around the lesion site. Double nuclei are visible in some cells. The thickness of the RPE cell layer is revealed in the vertical rectangle at the right and a deep capillary is visible within the lesion in the horizontal rectangle at the top (white arrow head). There is no adhering retinal material above the RPE, indicating that removal of the retina is complete. The scale bar represent 100 μm. The ganglion cell layer (GCL), the inner nuclear layer (INL), the inner plexiform layer (IPL), the region of photoreceptor degeneration (Degeneration Zone), and the sclera (Sc) are denoted.

Mice given laser treatment were anesthetized as above, and pupils dilated with 1% tropicamide (Akorn, Inc., Buffalo Grove, IL). For optimal viewing of the retina and placement of laser lesions, corneal optics were negated by a coverslip adhered with a drop of 2.5% methylcellulose on the mouse cornea. To initiate retinal CNV, three laser spots (diameter 50 μm; duration 100 ms; energy 150 mW) were administered to each fundus using a Novus Spectra ophthalmic 532-nm diode laser (Lumenis, Inc., Santa Clara, CA) mounted on a slit lamp (Model SL-07; Topcon, Inc., Tokyo, Japan). The superior, nasal-inferior, and temporal-inferior fundus regions each received one lesion. Lesions were placed between two to three optic disc diameters from the optic nerve and between the retinal vessels. Inclusion criteria for successful laser-induced CNV were the formation of a bubble immediately after laser application, indicating penetration of Bruch’s membrane (Figure 1), and absence of subretinal hemorrhage. Lesions violating these criteria were excluded from the study. In total, only four lesions were rejected for lack of bubble, and no single mouse had more than one rejected lesion. After full recovery from anesthesia, mice were returned to the animal colony.

### Fluorescein angiography

In vivo fundus fluorescein angiography of CNV lesions was performed 7 and 14 days post laser treatment using a Spectralis® HRA+OCT imaging system (Heidelberg Engineering, Inc., Vista, CA) equipped with high-resolution spectral domain optical coherence tomography and a confocal scanning laser ophthalmoscope. Mice were anesthetized and zero diopter contact lenses (Veterinary Specialty Products, Whitchurch, UK) were applied to improve image quality and protect corneas from desiccation. Following ip injection of 20 μl of 25% fluorescein (Hub Pharmaceuticals, Rancho Cucamonga, CA), angiograms were captured at approximately 0.5, 2, and 6 min post fluorescein injection.

### Immunohistochemistry and microscopy of choroid flat mounts

Mice were sacrificed at 15 days post laser to allow fluorescein from the 14-day angiography to dissipate. Eyes were enucleated, corneas slit, and eyes fixed overnight (4 °C) with 4% paraformaldehyde (Electron Microscopy Sciences, Hatfield, PA) in PBS (potassium phosphate monobasic, 10.59 mM; sodium chloride, 1551.72 mM; sodium phosphate dibasic, 29.66 mM; without calcium chloride or magnesium chloride; pH 7.4; GIBCO, St. Louis, MO). After fixation, anterior segments and retinas were removed. The remaining eyecups were devoid of adhering retinal material (Figure 1C). These were washed in PBS (3×10 min), then permeabilized and blocked with 2% normal donkey serum (Sigma, St. Louis, MO) and 1% Triton-X (Invitrogen, Carlsbad, CA) in PBS (3×10 min). Eyecups were then incubated overnight (4 °C) with primary antibodies, 2% normal donkey serum (Sigma-Aldrich, St. Louis, MO), and 0.1% Triton-X in PBS. After primary labeling, eyecups were washed in 2% normal donkey serum and 0.1% Triton-X in PBS (3×10 min), then incubated overnight (4 °C) with fluorescent secondary antibodies and probes. Following a final wash with PBS (3×10 min), labeled eyecups were flattened with four peripheral radial cuts and coverslipped with ProLong Gold anti-fade medium (Invitrogen), forming a choroid flat mount. Antibodies and probes used were CD11b (20 μg/ml; AbD Serotec, Raleigh, NC), detected with AlexaFluor® 488 (5 μg/ml; Invitrogen); isolectin B_4_ (IB_4_) from *Griffonia simplicifolia* conjugated to AlexaFluor® 568 (25 μg/ml; Invitrogen); Hoechst 33,258 (10 μg/ml; Invitrogen); and phalloidin (to detect f-actin) conjugated to AlexaFluor^®^ 488 (5 units/ml; Invitrogen).

Flat mounts were imaged on a Zeiss LSM-510 Meta laser confocal microscope using Zeiss objectives (Zeiss, Thornwood, NY). Tiled composites, or mosaics, were imaged using a Plan-Neofluar 10X/0.3 numerical aperture (NA) objective. Three-dimensional volumes were imaged using a Plan-Neofluar 40X/1.3 NA Oil DIC, Plan-Apochromat 63X/1.4 NA Oil DIC, or Plan-Apochromat 100X/1.4 NA Oil DIC objective. Optical slice thickness was equal across all channels and z-step intervals satisfied Nyquist sampling. Fluorophores were visualized as follows (excitation; emission): AlexaFluor® 568 (543 nm; 560–615 nm), AlexaFluor® 488 (488 nm; 505–550 nm), and Hoechst (405 nm; 420–490 nm).

To detail the effect of the laser on the retina, tissue was collected at 14 days post treatment and prepared for conventional histological analysis. Briefly, the corneas were slit and the eyes fixed in glutaraldehyde (2%) and paraformaldehyde (2%) in sodium cacodylate buffer (0.1 M) overnight. The cornea, iris, and lens were removed from each eye and fixation continued for one additional hour. Eyecups were rinsed in buffer and then postfixed in osmium tetroxide (1% in 0.1 M sodium cacodylate) for one hour, rinsed again, and dehydrated through an ethanol series to acetone. Infiltration and embedding occurred in an Embed-812/araldite epoxy mixture. Serial, 1 µm thick plastic sections were obtained through the laser lesions. These were contrasted with toluidine blue (1%) in 1% sodium borate and viewed by conventional light microscopy. Images were captured on a Nikon Optiphot-2 microscope (200X magnification) with a Nikon DS-Ri1 digital camera and displayed with NIS-Elements BR 3.00 software (Figure 1A,B).

### Leakage analysis

**Figure 2:**
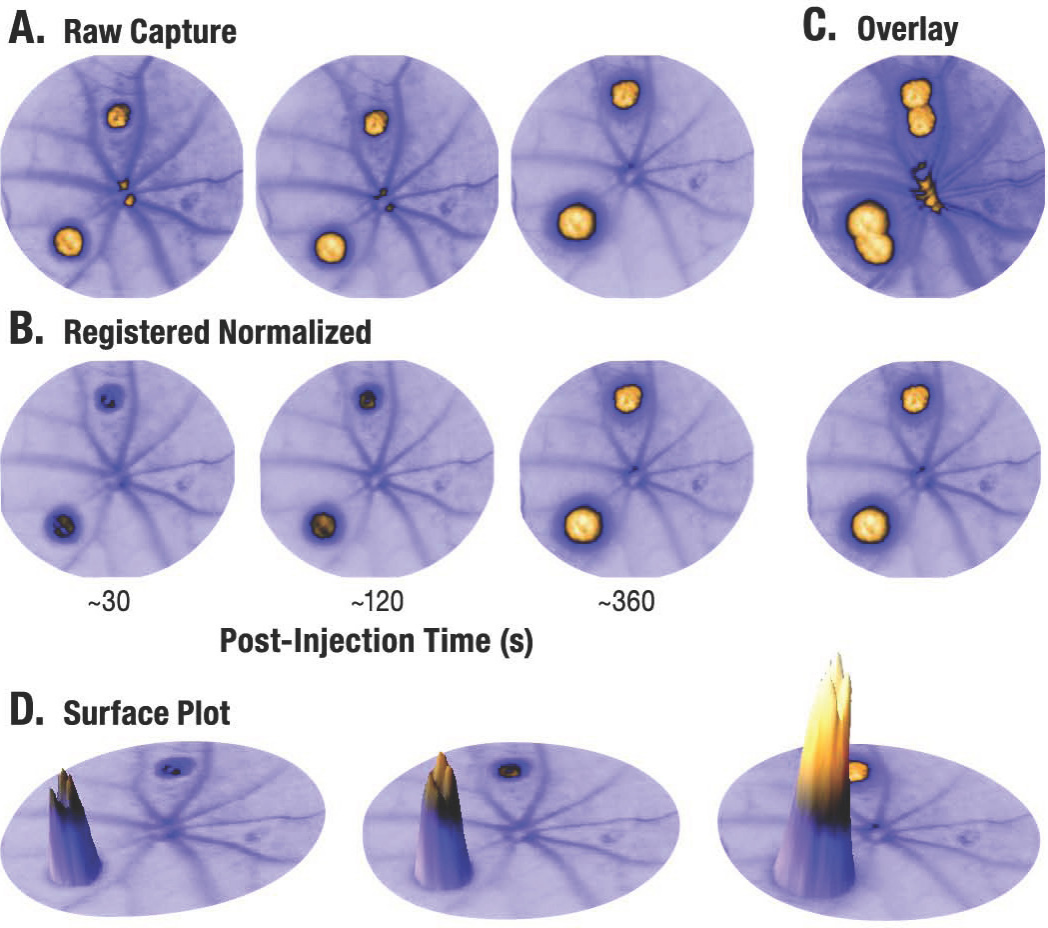
Angiogram processing. Raw images in an angiogram time series are captured unaligned and with arbitrary gain values. **A**: Gain values are reduced over time to accommodate increasing lesion intensity, thereby concealing the majority of fluorescein changes between phases. **B**: Registered and normalized angiograms reveal a clear time-dependent accumulation of fluorescein within lesions. Note that vascular fluorescein intensity remains stable across phases. **C**: An overlay of the phases illustrates the accuracy of registration using scale invariant feature transform (SIFT) alignment. **D**: A surface plot visualizes the integrated density (area multiplied by intensity) of lesion regions of interest (ROIs). Fluorescein intensity is plotted along the Z dimension and the ROI boundary lies on the XY plane. The volume of the extruded cone is equivalent to integrated density, which clearly increases over time.

Raw images of an angiography series were preprocessed (Figure 2) and lesion regions of interest (ROIs) were selected using an eight connected region growing method with manual subtraction of outlying regions. Leakage was measured as the area, mean intensity, and integrated density of each lesion at each phase and the exact fluorescein time of the phase recorded.

### Analysis of choroidal neovascularization area

Based on the observed low variance of IB_4_ labeling along the Z-axis during our previous CNV analysis [6], we investigated CNV in the current study using area rather than volume. The CNV area was measured from the IB_4_ channel of choroid flat mount mosaics in Fiji by defining ROIs with eight connected region growing and the manual elimination of outlying pixels.

### Analysis of microglia

Microglia were identified by CD11b. Using a custom programmed macro in Fiji, sectors (origins at the optic nerve) were circumscribed ± 45 degrees about the centroid of each lesion in choroid flat mount mosaics. The radius of each lesion sector was 1080 μm, producing an area of 915,391 μm^2^. Microglia that did not overlap with the CNV lesion, hereafter referred to as peripheral microglia, were counted within each sector and the distance to the nearest lesion edge measured to assess their spatial distribution.

Microglia were segmented and their morphology assessed in two-dimensional (2D) and three-dimensional (3D) space. For 2D space, maximum intensity projections of the XY planes were used. ImageJ’s built-in analysis for circularity (shape descriptors form factor), solidity, convexity, area, and perimeter were measured in 2D, and 3D equivalents were measured using our custom 3D shape plugin. Fractal dimension was measured in 2D and 3D using the Fractal Count plugin [11].

### Statistical methods

Leakage, CNV, microglia count, and microglia spatial distribution were each analyzed separately using a mixed-model procedure [12] in SAS v9.1 (SAS Institute Inc., Cary, NC). Observations and denominator degrees of freedom (obs, den df) were as follows: leakage (168, 111); CNV area (50, 48); microglial count (33, 31); and microglial spatial distribution (965, 933). Treatment effects are represented by least squares and means±standard error reported p values are adjusted using the simulate option to increase robustness.

Analysis of microglial morphology was performed using the R statistical programming language. Treatment effects were assessed both nonparametrically using the Kruskal–Wallis and Wilcoxon tests and parametrically by a single-factor analysis of variance. Prior to analysis of variance testing, morphological measures rejected by the Shapiro-Wilk test of normality (α=0.1) were iteratively transformed using the method described by Goerg [13,14]. Data are presented as the transformed mean ± standard error of the mean; reported p values were White corrected [15].

## Results

### Choroidal neovascularization

**Figure 3:**
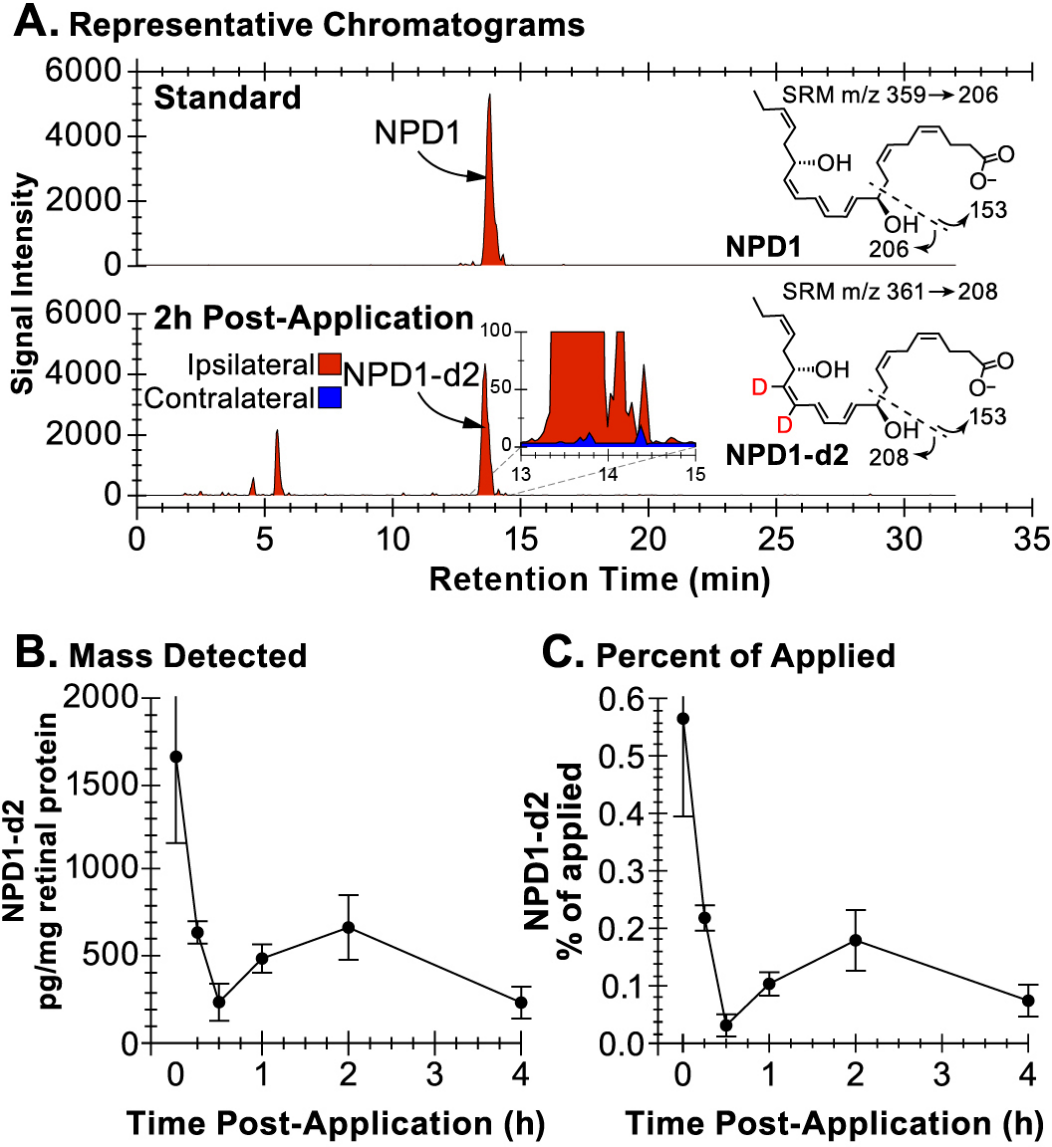
Mass spectrometry and neuroprotectin D1 delivery to retina by topical corneal application. **A**: Sample chromatograms illustrate the presence of deuterated neuroprotectin D1 (NPD1-d2) in ipsilateral retina 2 h after topical application, but not contralateral retina (bottom inset). Structural schematics of the selected reaction monitoring (SRM) for NPD1 (m/z 359 to 206) and NPD1-d2 (m/z 361 to 208) are shown at the right of each chromatogram. **B**: Retinal tissue concentration versus time post application of NPD1-d2 reveals a sustained peak near 2 h. The NPD1-d2 spike at 0 h most likely results from aqueous humor contamination of tissue samples due to the extraction method. **C**: NPD1-d2 concentration normalized to the mass of topically applied NPD1-d2. For Control, n=87; for NPD1, n=81; for total observations, n=168; and degrees of freedom = 111.

The retinal bioavailability of topically applied NPD1 was assessed and found to have peak concentrations 2 h after application (Figure 3); no measurable quantity of nondeuterated NPD1, representing endogenous synthesis, was detected. It should be noted that NPD1 day 2 was applied in a 97.5% ethanol solution, which may have affected the aqueous humor dynamics of the mouse eye. From eye grooming behavior, it was inferred that mice experienced temporary irritation after both NPD1 and vehicle treatments, but otherwise tolerated them well; no corneal desiccation or cataract formation was observed in any eye during the treatment schedule.

**Figure 4:**
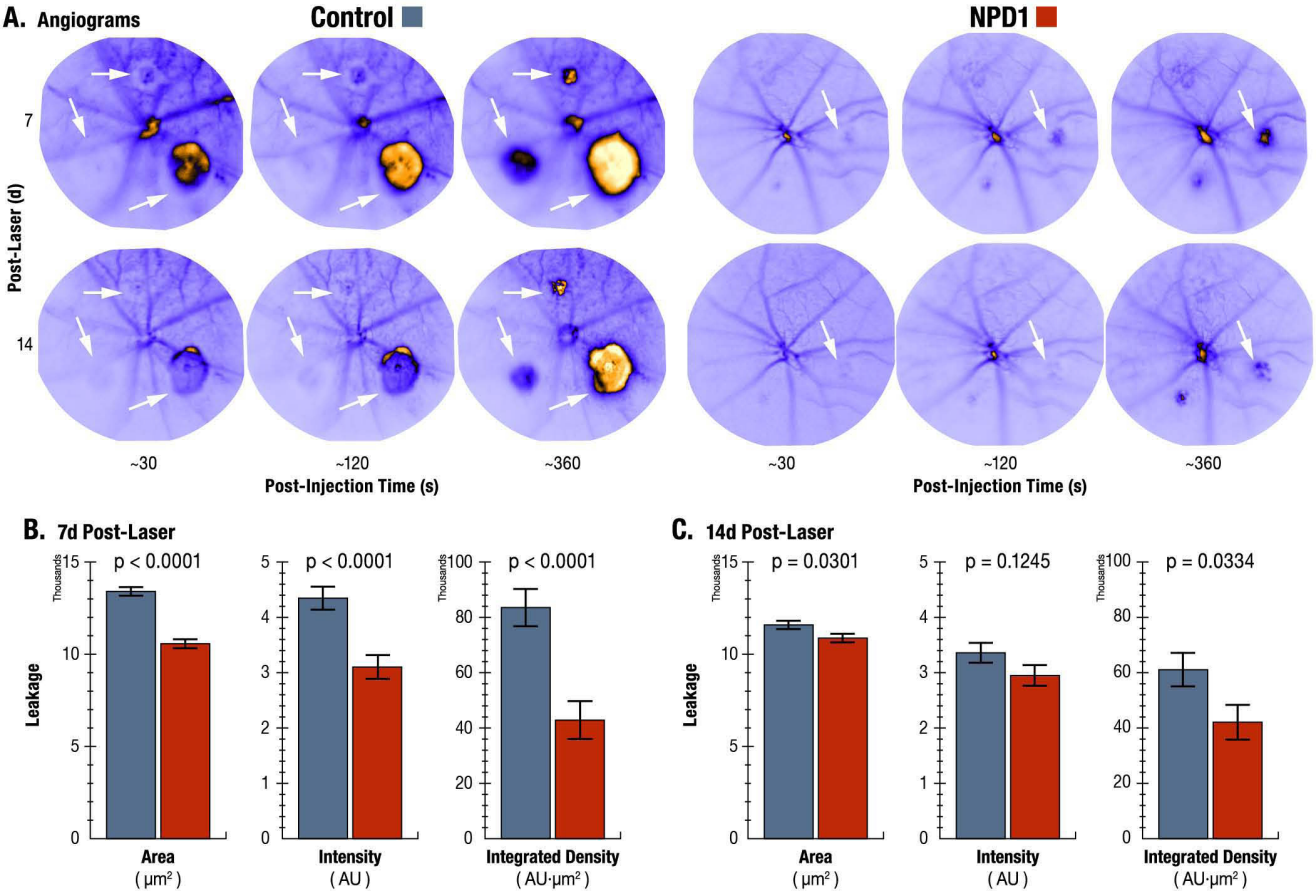
Comparison of fluorescein leakage. **A**: Representative fluorescein angiograms of control and neuroprotectin D1 (NPD1)-treated eyes from the same animal at 7 and 14 days post laser are shown. The arrowheads illustrate an annular fluorescein void surrounding choroidal neovascularization (CNV) lesions. Increases of fluorescein intensity with time are clearly evident in both treatments, but are significantly higher in control lesions than in NPD1 lesions. **B**: The results of quantitative image analysis at 7 days post laser indicate that NPD1 treatment significantly reduced the area, intensity, and integrated density of leakage. **C**: At 14 days post laser, 1 week after cessation of treatment, NPD1-induced reductions in leakage area and integrated density were sustained. For Control, n=87; for NPD1, n=81; for total observations, n=168; and degrees of freedom = 111.

Fluorescein angiography revealed striking differences between CNV lesions in control and NPD1-treated animals (Figure 4A). Leakage in NPD1-treated eyes was significantly lower (p<0.0001) than in controls at 7 days post laser (Figure 4B). By 14 days post laser, two of the three leakage measures remained significantly lower (p<0.05) in the treatment group (Figure 4C).

**Figure 5:**
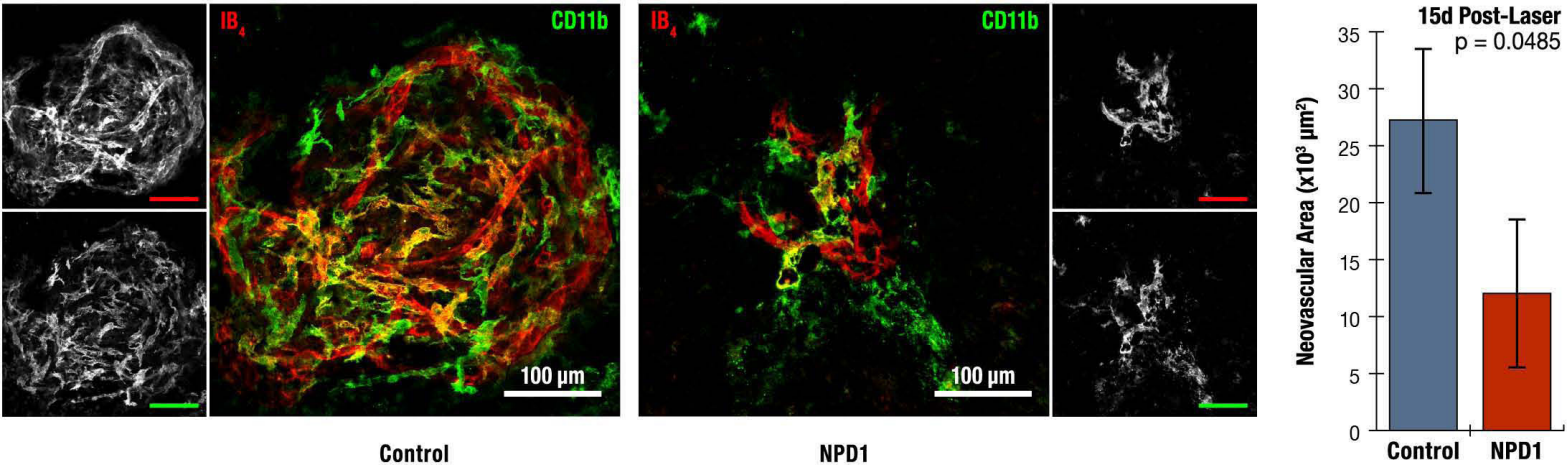
Comparison of neovascularization in control and neuroprotectin D1-treated eyes. Choroidal neovascular lesions from control and neuroprotectin D1 (NPD1)-treated eyes of the same animal were visualized using isolectin B_4_ *Griffonia simplicifolia* (red) and CD11b (green). Control lesions demonstrate extensive ramification of both vascular endothelia (red) and monocytes (green). Neovasculature is drastically attenuated in NPD1-treated lesions. Quantitation of the lesion areas revealed a 56% reduction in neovascularization due to topical application of NPD1. For Control, n=26; for NPD1, n=24; for total observations, n=50; and degrees of freedom =48.

Immunohistochemical labeling of vascular endothelial cells with isolectin B_4_ also demonstrated striking differences between treatment groups. At 15 days post laser, the area of labeled neovasculature in the NPD1-treated group was significantly lower (p<0.05) at approximately half that of the control group (Figure 5). Despite measuring the area rather than the volume, this level of reduction was similar to our previous results [6]. It should be noted that there were some yellow/orange labeled cells, which may indicate colocalization of reactivity for isolectin B_4_ and immunoreactivity for CD11b. It has been reported that microglia can be labeled with isolectin B_4_.

### Microglia

**Figure 6:**
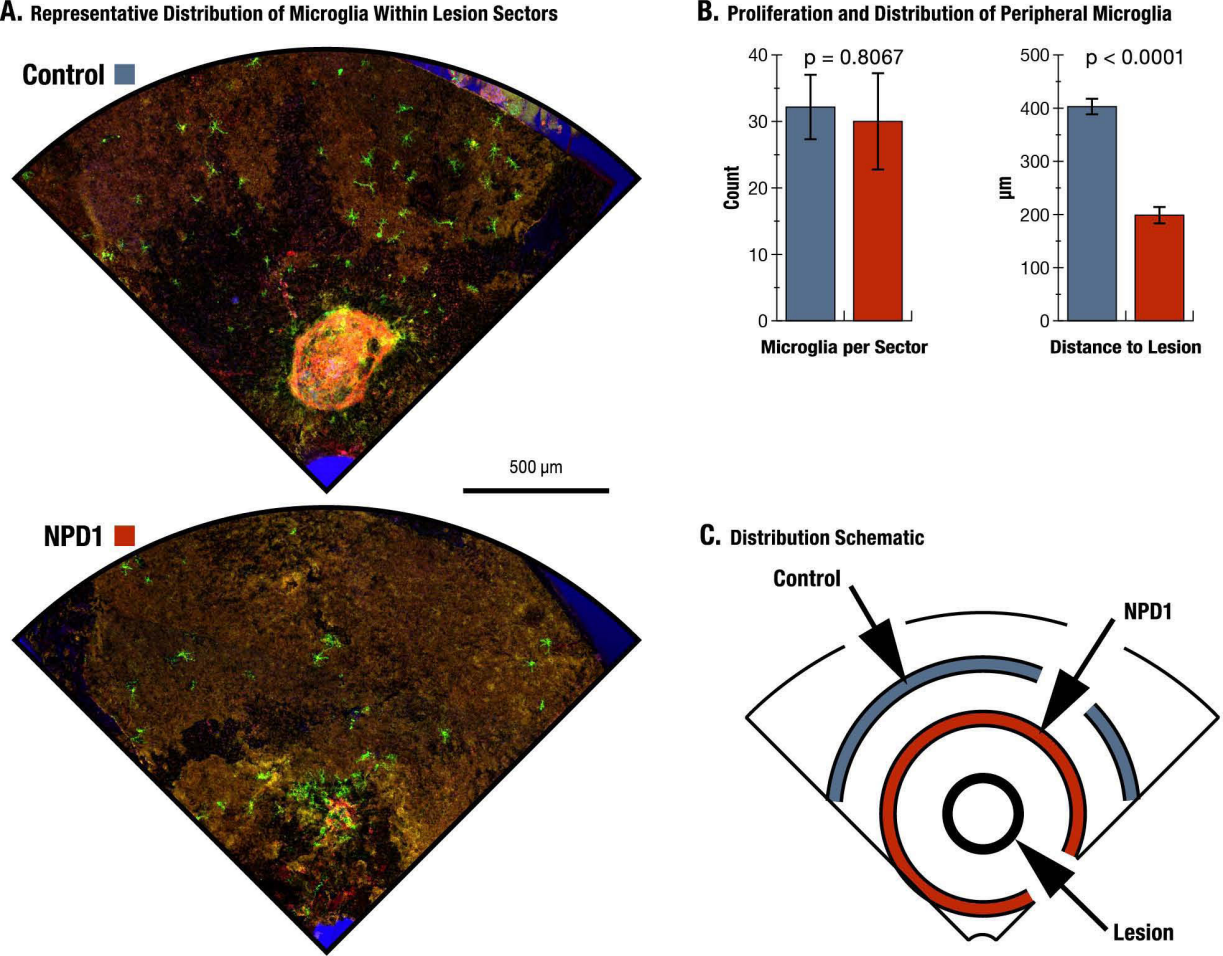
Microglial distribution in control and neuroprotectin D1-treated eyes. **A**: Lesion sectors from control and neuroprotectin D1 (NPD1)-treated eyes of the same animal highlight the differences in microglial distribution between treatments. **B**: The quantity of peripheral microglia per lesion sector was similar between treatments, but the spatial distribution was significantly different, with NPD1-treated microglia clustering 51% closer to the lesion perimeter than controls. The microglia count per sector is as follows: for Control, n=17; for NPD1, n=16; for total observations, n=3; and degrees of freedom = 31. The microglia distribution is as follows: for Control, n=503; for NPD1, n=462; for total observations, n=965; and degree of freedom = 933. **C**: An idealized schematic of a lesion sector illustrates a central lesion (black ring) with a ring for each treatment, representing the mean distance of microglia to the lesion perimeter.

Microglia peripheral to the CNV lesions were analyzed to assess density and recruitment effects induced by NPD1 (Figure 6). No difference in microglia counts was found between control and NPD1; control eyes had 24±2 microglia per lesion sector, while NPD1 eyes had 23±4 (Figure 6B). The spatial distributions within lesion sectors, however, were not homogenous, and revealed a significant difference between treatments. Microglia in control eyes were situated at an average of 344 μm from the CNV lesions, while NPD1 significantly reduced (p<0.0001) this distance by ~40% to 208 μm (Figure 6B).

**Figure 7:**
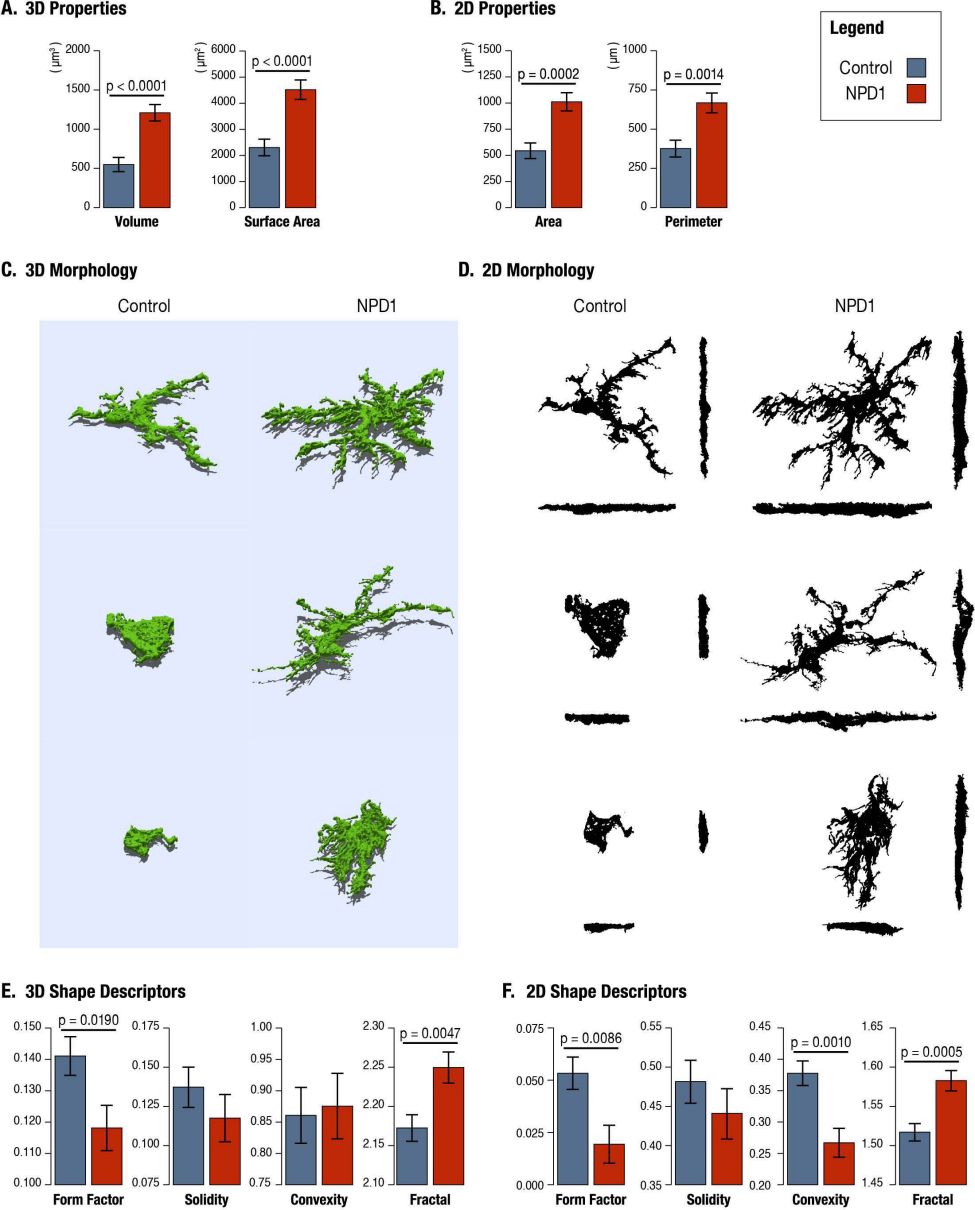
Quantitative analysis of microglial morphology. Examination of the properties and shape of microglia in two dimensions (2D) and three dimensions (3D) reveals significant differences between treatments. **A**: Neuroprotectin D1 (NPD1)-treated microglial cell areas and perimeters are significantly greater than controls. **B**: Microglial volumes and surface areas are significantly larger with NPD1 treatment. **C,D**: Morphological differences between treatments are readily apparent from representative maximum intensity projections (**C**) and 3D renderings (**D**); insets tabulate the values of the respective 2D and 3D shape descriptors (S: solidity). For 2D projections, the XY projection is shown centrally, with the XZ projection below and the YZ projection to the right. **E**: Quantitative analysis of shape descriptors using classic 2D maximum XY projections reveal significant differences in form factor (F), convexity (C), and fractal dimension (D) between treatments when analyzed using 2D maximum XY projections. The 3D shape descriptors reveal that NPD1 treatment significantly reduces the form factor and significantly increases the fractal dimension. For Control, n=33; for NPD1, n=24; for total observations, n=50; and degrees of freedom = 55.

Morphologically, microglial cells in the NPD1 treatment group had more than double the volume (p<0.0001), and a near twofold increase in surface area (p<0.0001; Figure 7A). Much of this cellular change occurred orthogonal to the Z-axis, as maximum intensity XY projections also revealed significant increases in analogous measures; the area increased 1.9-fold and the perimeter increased 1.8-fold (p=0.0002 and p=0.0014, respectively; Figure 7B).

**Figure 8:**
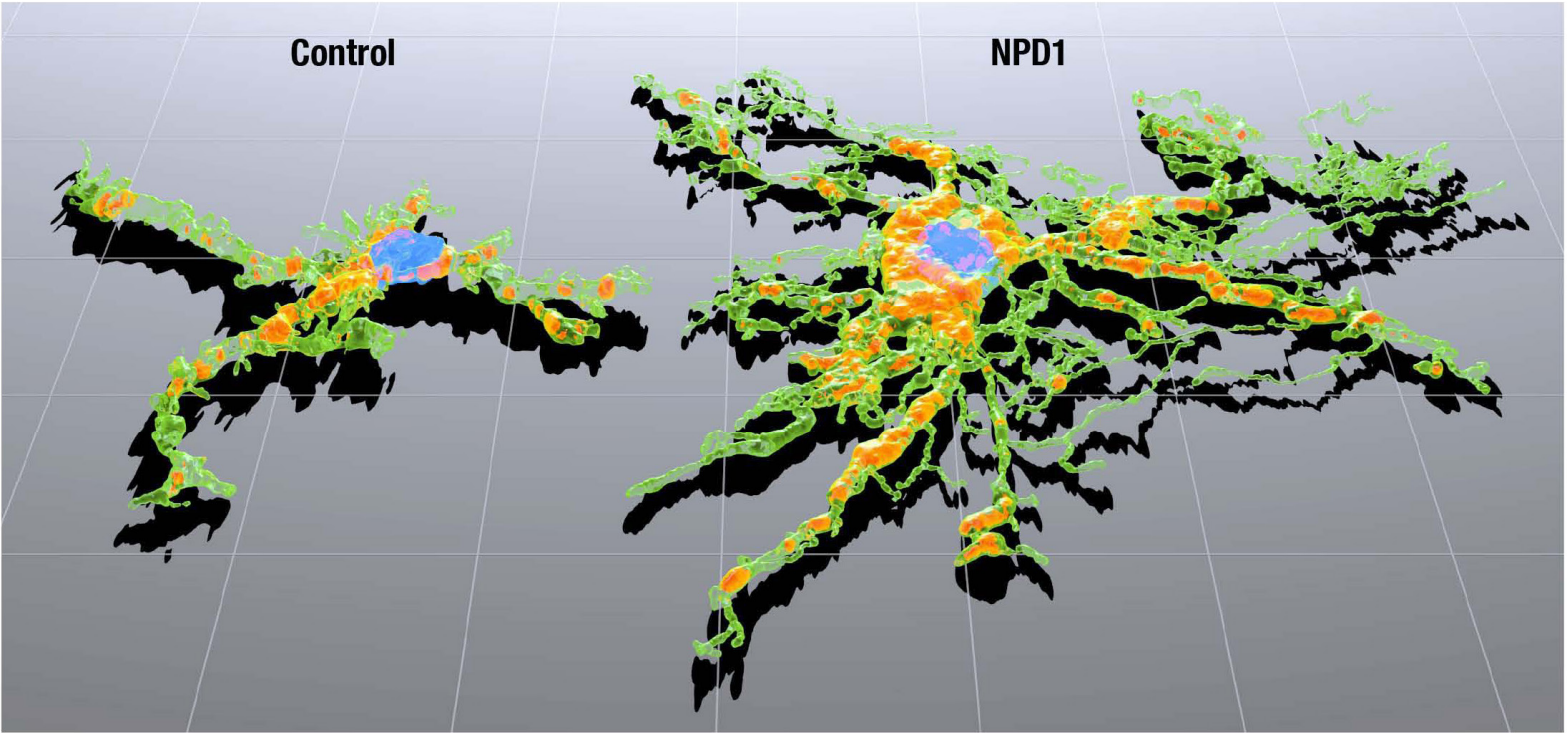
Comparison of representative microglia rendered in three dimensions. Microglia from control and neuroprotectin D1 (NPD1)-treated eyes are shown adjacent to each other and rendered with a three-dimensional (3D) perspective to illustrate the increased size and ramification promoted by NPD1 treatment. The grid size is 25 μm.

We further investigated the continuum of alterations in reactive microglia using quantitative shape descriptors. The form factor expresses the spherical (circular for 2D) shape of an object. In both 3D and 2D, the form factor of the peripheral microglia was lower in the NPD1 group, indicating that microglia in the control group were more spherical and rounded (Figure 7C-F). Solidity, which conveys the spatial density of an object, was also lower in both 3D and 2D with NPD1 treatment, but not significantly different from controls (Figure 7E,F). Convexity was lower in the NPD1 group in 2D but not 3D (Figure 7E,F). Unlike the previous shape parameters, which are inherently linked to the image magnification, the fractal dimension evaluates the rate of change in the surface area (perimeter in 2D) with regard to scale and can be simply thought of as the roughness of the cell surface. NPD1 significantly increased the fractal dimension in both 3D and 2D (Figure 7E,F), implying that there were rougher cell surfaces than in control microglia. Visually, the differences in size and complexity between microglia were easily seen in the 3D rendering of representative microglia from control and NPD1 treatments (Figure 8).

## Discussion

NPD1 is well suited for topical delivery due to its lipophilicity and low molecular weight, both of which increase permeability in the cornea, conjunctiva, and retinal pigment epithelium (RPE) [16]. Thus, we investigated the efficacy of topically applied NPD1 to attenuate CNV. Because NPD1 is a potent mediator of neuroprotection and inflammatory resolution, it is tightly regulated, and in the absence of an initiating trigger, little to no NPD1 is synthesized. With topical eye drops being the least invasive ocular drug delivery method, NPD1 synthesis was not an expected outcome of our treatment process. The absence of endogenous NPD1 in our bioavailability assessment accords with this expectation. Despite the strict governance of NPD1 synthesis and release, exogenously applied NPD1-d2 was indeed present in retinal tissue at detectable levels. Considering our method of retina extraction, some degree of contamination by aqueous humor is assured, resulting in the high concentration at time zero. The decrease at plus 15 and 30 min suggests that the labeled NPD1 had moved out of the aqueous humor and into more distal tissues. A slower second NPD1-d2 peak at 2 h, however, supports the clear presence of NPD1 in retinal tissue. Our results demonstrate proof of principle that NPD1 can reach retinal tissue and that topical application is a viable delivery route for NPD1. Importantly, when diluted to therapeutic concentrations in saline, topical NPD1 treatment is well tolerated by the cornea.

One week after inducing CNV, NPD1 treatment significantly reduced leakage by approximately half compared to untreated controls. While NPD1-treated lesions in the second week did exhibit one-third less leakage than controls, the difference was not statistically significant, as the control lesions demonstrated a self-resolving leakage reduction of 27% from the first week. Considering that treatment was discontinued after the first week, these results demonstrate that NPD1 attenuates leakage before the normal resolution process observed in controls. As leakage reductions ceased when treatment did, it is possible that a continuous treatment paradigm may result in further leakage improvements. Topical NPD1 treatment also reduced the neovascular area by half. The leakage and CNV results observed with topical application correspond well to our previous study using ip delivery [6] and corroborate our previous findings that NPD1, whether applied topically or injected ip, attenuates CNV and associated leakage.

The ability of microglia to activate, proliferate, and migrate to sites of inflammation are requisite steps in the resolution of neuroinflammatory diseases. Here, we investigated the ability of NPD1 treatment to modulate microglial activation and recruitment. Our results showed similar quantities of microglia in both treatments. When we examined the spatial distribution of microglia, however, we found that microglia in NPD1 treated eyes were half the distance to lesions observed in control eyes. This suggests that NPD1 signaling facilitates microglial recruitment. In response to injury, microglia polarize toward the lesion and shield the injured area with an accumulation of microglial extensions [17]. The distribution pattern of microglia in NPD1-treated eyes is reminiscent of this shielding behavior. It has been observed in studies on hippocampal slices that activated microglia appear to wrap around capillaries to contain leakage sites [18]. Unfortunately, the limited immunolocalization of the present study did not permit visualization of this interaction. Here, it appears that microglia did not achieve this intimacy under either condition. However, since polarization, shielding, and migration are dependent on process motility [17-19], these results do suggest that NPD1 may enhance the dynamics of microglial processes responding to acute local injury signals.

Next, we evaluated differences in activation between treatments. As the functional phenotype of microglia is closely tied to their morphology, we used morphological shape as a proxy to assess differences in microglial activation. In normal healthy retinas, microglia are stratified into the outer and inner plexiform layers, as well as the ganglion cell layer [20,21]. In the surveillance state [22], originally termed “resting,” the resident microglia exhibit substantial ramification within their stratum [20]. Microglia present in choroid flat mounts are thus activated, but our results indicate that the degree of microglial activation differs between treatments. Indeed, it has been suggested that microglia may achieve various levels of activation under diverse conditions, in distinct regions, and in different tissues [23]. Morphologically, control microglia had smoother cell surfaces (lower fractal dimensions) and a more rounded form (higher form factor), and were half the size of microglia in the NPD1 group; all of these features are consistent with the morphology of amoeboid microglia [24,25]. Conversely, NPD1 treatment resulted in a more ramified morphology. This outcome may be simply a result of the CNV reduction induced by NPD1. Alternatively, NPD1 signaling may target microglia to affect the outcome of CNV. It is not yet clear whether microglial activation is caused by the initial damage from the laser treatment, or alternatively, because of some aspect of the development of the choroidal vessels. These neovessels could potentially leak, and instead provide signals secondary to the laser damage for activation of the microglia. Regardless, the lesion-associated microglia in the NPD1-treated eyes develop a highly ramified morphology that resembles microglia in their postactivated surveillance state, suggesting a condition more associated with resolution [23].

In our previous study, NPD1 was injected intraperitoneally 1 h before and 1, 3, 5, and 7 days following the initiation of the laser-induced CNV, and immunolabeling was performed on flat mount preparations 1 and 2 weeks following laser application. While NPD1 was administered only during the first week, we found continual reduction of leakage throughout the second week, indicating that NPD1 had an ongoing and direct effect on the underlying pathogenic process. Endothelial cell growth was affected in a similar manner; cell volume was significantly lower at the end of the first week and continued to decline during week 2 [6]. We noted similar results throughout the first week in this study, but observed a marked slowing of the decline during the following week, perhaps because of differing retinal NPD1 concentrations resulting from different modes of delivery.

We have suggested that the NPD1-induced reduction of laser-CNV leakage may occur through inhibition of cyclooxygenase 2 (COX-2) expression and nuclear factor kappa B (NF-κB) activation [9], and have shown that COX-2 and vascular endothelial growth factor (VEGF) expression increase when retinal choroidal cells are stimulated with interleukin (IL)-1β [26]. Moreover, NPD1 downregulates IL-1β and tumor necrosis factor (TNF)-α gene expression [27], both of which are known to increase permeability of the pericyte/endothelial cell complex [28]. Accordingly, NPD1-induced inhibition of CNV-associated leakage may result from the reduction of IL-1β and TNF-α expression, as well as the inhibition of NF-κB, a common downstream transcription factor in both pathways. Decreased permeability (i.e., vascular leakage) at the pericyte/endothelial cell complex and a reduction in VEGF expression would result from this, reducing the growth and permeability of endothelial cells.

NPD1 potently promotes cell survival. We have demonstrated that RPE cells are protected from oxidative stress– and inflammation-induced apoptosis by NPD1 [7,8]. Promoting RPE cell survival may encourage the resynthesis of Bruch’s membrane and help to maintain RPE tight junctions, resulting in the reestablishment of an intact subretinal space that is protected from neovascular signaling with the choroid. Moreover, neuronal and glial cell inflammatory Aβ-42-triggered apoptosis and optic nerve damage–induced retinal ganglion cell death are inhibited by NPD1 [27,29], indicating that photoreceptors and Müller glial cells may be protected from CNV inflammatory signaling by NPD1.

In the present study, laser-induced CNV created large neovascularized wound sites with associated microglia, but did little to affect the distribution of microglia that had migrated into the subretinal space. In contrast, treatment with NPD1 exerted neuroprotective action at the laser damage sites, decreasing the volume of the wound site and its neovascular tissue and stimulating microglial migration from the subretinal space to accumulate at the lesions.

It was recently demonstrated that activated microglia can respond to tissue damage by expressing TNF-α [30]. However, we have shown that NPD1 downregulates proinflammatory substances such as IL-1β and TNF-α [27], and the present study suggests that NPD1 treatment blocked the proinflammatory effects of microglia but promoted both tissue aggregation of microglia at the damage site and an anti-inflammatory response. In addition, as in the previous study, NPD1 administration occurred only during the first week, but analysis after 2 weeks demonstrated marked differences in microglial distribution and morphology when compared to untreated retinas undergoing CNV, again suggesting that the effect of NPD1 on the pathoangiogenesis of laser-induced CNV may be long lasting.

These observations suggest that NPD1 may trigger multiple effects on activated microglia, resulting in the promotion of anti-inflammatory responses. Taken together, these results strongly suggest that NPD1 signaling exerts multiple effects on the cross-talk between microglia and the retina/RPE/choroidal complex.

In the present study, we have demonstrated that topical application of NPD1 to the eye surface is well tolerated, reaches retinal tissue, and ameliorates experimental CNV. Importantly, this study corroborates our previous findings of NPD1 attenuating CNV and affirms that NPD1 signaling imparts significant neuroprotection and is capable of resolving neuroinflammatory conditions. The discovery of the redistribution and ramification of microglia concomitant with NPD1-attenuated CNV suggests that NPD1 signaling may function to initiate phenotypic changes within microglia. Since microglia are crucial participants in neurodegenerative diseases, the discovery of microglia as potential targets of NPD1 signaling warrants further investigation.

## References

[1] Caputo M, Zirpoli H, Di Benedetto R, De Nadai K, Tecce MF (2011). Perspectives of choroidal neovascularization therapy.. Curr Drug Targets.

[2] Combadière C, Feumi C, Raoul W, Keller N, Rodéro M, Pézard A, Lavalette S, Houssier M, Jonet L, Picard E, Debré P, Sirinyan M, Deterre P, Ferroukhi T, Cohen SY, Chauvaud D, Jeanny JC, Chemtob S, Behar-Cohen F, Sennlaub F (2007). CX3CR1-dependent subretinal microglia cell accumulation is associated with cardinal features of age-related macular degeneration.. J Clin Invest.

[3] Streit WJ (2002). Microglia as neuroprotective, immunocompetent cells of the CNS.. Glia.

[4] Buschini E, Piras A, Nuzzi R, Vercelli A (2011). Age related macular degeneration and drusen: neuroinflammation in the retina.. Prog Neurobiol.

[5] Gupta N, Brown KE, Milam AH (2003). Activated microglia in human retinitis pigmentosa, late-onset retinal degeneration, and age-related macular degeneration.. Exp Eye Res.

[6] Sheets KG, Zhou Y, Ertel MK, Knott EJ, Regan CE, Elison JR, Gordon WC, Gjorstrup P, Bazan NG (2010). Neuroprotectin D1 attenuates laser-induced choroidal neovascularization in mouse.. Mol Vis.

[7] Mukherjee PK, Marcheselli VL, Barreiro S, Hu J, Bok D, Bazan NG (2007). Neurotrophins enhance retinal pigment epithelial cell survival through neuroprotectin D1 signaling.. Proc Natl Acad Sci USA.

[8] Mukherjee PK, Marcheselli VL, de Rivero Vaccari JC, Gordon WC, Jackson FE, Bazan NG (2007). Photoreceptor outer segment phagocytosis attenuates oxidative stress-induced apoptosis with concomitant neuroprotectin D1 synthesis.. Proc Natl Acad Sci USA.

[9] Marcheselli VL, Hong S, Lukiw WJ, Tian XH, Gronert K, Musto A, Hardy M, Gimenez JM, Chiang N, Serhan CN, Bazan NG (2003). Novel docosanoids inhibit brain ischemia-reperfusion-mediated leukocyte infiltration and pro-inflammatory gene expression.. J Biol Chem.

[10] Petasis NA, Yang R, Winkler JW, Zhu M, Uddin J, Bazan NG, Serhan CN (2012). Stereocontrolled total synthesis of neuroprotectin D1/protectin D1 and its aspirin-triggered stereoisomer.. Tetrahedron Lett.

[11] Henden PC, Bache-Wiig J. *Fractal Count Plugin* Programvareverkstedet ved Norges Teknisk Naturvitenskapelige Universitet; 2005. Available at: http://www.pvv.org/~perchrh/imagej/fractal.html

[12] Milliken GA, Johnson DE. *Analysis of Messy Data Volume 1: Designed Experiments, Second Edition* 2nd ed. Boca Raton, FL: Chapman and Hall/CRC; 2004.

[13] Goerg GM. The Lambert Way to Gaussianize skewed, heavy tailed data with the inverse of Tukey's h transformation as a special case. *arXiv* 2010;1010.2265(4):1–40. Available at: http://arxiv.org/abs/1010.2265 Accessed November 11, 2011. 10.1155/2015/909231 PMC4562338 26380372

[14] Goerg GM, Lambert W (2011). Random Variables - A New Family of Generalized Skewed Distributions with Applications to Risk Estimation.. Annals of Applied Statistics.

[15] Long JS, Ervin LH (2000). Using heteroscedasticity consistent standard errors in the linear regression model.. Am Stat.

[16] Edelhauser HF, Rowe-Rendleman CL, Robinson MR, Dawson DG, Chader GJ, Grossniklaus HE, Rittenhouse KD, Wilson CG, Weber DA, Kuppermann BD, Csaky KG, Olsen TW, Kompella UB, Holers VM, Hageman GS, Gilger BC, Campochiaro PA, Whitcup SM, Wong WT (2010). Ophthalmic drug delivery systems for the treatment of retinal diseases: basic research to clinical applications.. Invest Ophthalmol Vis Sci.

[17] Nimmerjahn A, Kirchhoff F, Helmchen F (2005). Resting microglial cells are highly dynamic surveillants of brain parenchyma in vivo.. Science.

[18] Stence N, Waite M, Dailey ME (2001). Dynamics of microglial activation: a confocal time-lapse analysis in hippocampal slices.. Glia.

[19] Liang KJ, Lee JE, Wang YD, Ma W, Fontainhas AM, Fariss RN, Wong WT (2009). Regulation of dynamic behavior of retinal microglia by CX3CR1 signaling.. Invest Ophthalmol Vis Sci.

[20] Santos AM, Martín-Oliva D, Ferrer-Martín RM, Tassi M, Calvente R, Sierra A, Carrasco MC, Marín-Teva JL, Navascués J, Cuadros MA (2010). Microglial response to light-induced photoreceptor degeneration in the mouse retina.. J Comp Neurol.

[21] Lee JE, Liang KJ, Fariss RN, Wong WT (2008). Ex vivo dynamic imaging of retinal microglia using time-lapse confocal microscopy.. Invest Ophthalmol Vis Sci.

[22] Hanisch U-K, Kettenmann H (2007). Microglia: active sensor and versatile effector cells in the normal and pathologic brain.. Nat Neurosci.

[23] Kettenmann H, Hanisch U-K, Noda M, Verkhratsky A (2011). Physiology of microglia.. Physiol Rev.

[24] Soltys Z, Orzylowska-Sliwinska O, Zaremba M, Orlowski D, Piechota M, Fiedorowicz A, Janeczko K, Oderfeld-Nowak B (2005). Quantitative morphological study of microglial cells in the ischemic rat brain using principal component analysis.. J Neurosci Methods.

[25] Soltys Z, Ziaja M, Pawlínski R, Setkowicz Z, Janeczko K (2001). Morphology of reactive microglia in the injured cerebral cortex. Fractal analysis and complementary quantitative methods.. J Neurosci Res.

[26] Lukiw WJ, Ottlecz A, Lambrou G, Grueninger M, Finley J, Thompson HW, Bazan NG (2003). Coordinate activation of HIF-1 and NF-kappaB DNA binding and COX-2 and VEGF expression in retinal cells by hypoxia.. Invest Ophthalmol Vis Sci.

[27] Lukiw WJ, Cui J-G, Marcheselli VL, Bodker M, Botkjaer A, Gotlinger K, Serhan CN, Bazan NG (2005). A role for docosahexaenoic acid-derived neuroprotectin D1 in neural cell survival and Alzheimer disease.. J Clin Invest.

[28] Didier N, Romero IA, Créminon C, Wijkhuisen A, Grassi J, Mabondzo A (2003). Secretion of interleukin-1beta by astrocytes mediates endothelin-1 and tumour necrosis factor-alpha effects on human brain microvascular endothelial cell permeability.. J Neurochem.

[29] Qin Q, Patil KA, Gronert K, Sharma SC (2008). Neuroprotectin D1 inhibits retinal ganglion cell death following axotomy.. Prostaglandins Leukot Essent Fatty Acids.

[30] Roh M, Zhang Y, Murakami Y, Thanos A, Lee SC, Vavvas DG, Benowitz LI, Miller JW (2012). Etanercept, a widely used inhibitor of tumor necrosis factor-α (TNF-α), prevents retinal ganglion cell loss in a rat model of glaucoma.. PLoS ONE.

